# The golden hour of sepsis: An in-depth analysis of sepsis-related maternal mortality in middle-income country Suriname

**DOI:** 10.1371/journal.pone.0200281

**Published:** 2018-07-10

**Authors:** Lachmi R. Kodan, Kim J. C. Verschueren, Humphrey H. H. Kanhai, Jos J. M. van Roosmalen, Kitty W. M. Bloemenkamp, Marcus J. Rijken

**Affiliations:** 1 Department of Obstetrics and Gynaecology, Academical Hospital Paramaribo (AZP), Paramaribo, Suriname; 2 Department of Obstetrics, Division Women and Baby, Birth Centre, University Medical Center Utrecht, Utrecht, the Netherlands; 3 Julius Global Health, The Julius Centre for Health Sciences and Primary Care, University Medical Centre Utrecht, Utrecht, The Netherlands; 4 Department of Obstetrics, Leiden University Medical Centre, Leiden, the Netherlands; 5 Anton de Kom University, Paramaribo, Suriname; 6 Athena Institute, VU University Amsterdam, Amsterdam, the Netherlands; King's College London, UNITED KINGDOM

## Abstract

**Background:**

Sepsis was the main cause of maternal mortality in Suriname, a middle-income country. Objective of this study was to perform a qualitative analysis of the clinical and management aspects of sepsis-related maternal deaths with a focus on the ‘golden hour’ principle of antibiotic therapy.

**Methods:**

A nationwide reproductive age mortality survey was performed from 2010 to 2014 to identify and audit all maternal deaths in Suriname. All sepsis-related deaths were reviewed by a local expert committee to assess socio-demographic characteristics, clinical aspects and substandard care.

**Results:**

Of all 65 maternal deaths in Suriname 29 (45%) were sepsis-related. These women were mostly of low socio-economic class (n = 23, 82%), of Maroon ethnicity (n = 14, 48%) and most deaths occurred postpartum (n = 21, 72%). Underlying causes were pneumonia (n = 14, 48%), wound infections (n = 3, 10%) and endometritis (n = 3, 10%). Bacterial growth was detected in 10 (50%) of the 20 available blood cultures. None of the women with sepsis as underlying cause of death received antibiotic treatment within the first hour, although most women fulfilled the diagnostic criteria of sepsis upon admission. In 27 (93%) of the 29 women from which sufficient information was available, substandard care factors were identified: delay in monitoring in 16 (59%) women, in diagnosis in 17 (63%) and in treatment in 21 (78%).

**Conclusion:**

In Suriname, a middle-income country, maternal mortality could be reduced by improving early recognition and timely diagnosis of sepsis, vital signs monitoring and immediate antibiotic infusion (within the golden hour).

## Introduction

Sepsis is a major cause of severe maternal morbidity and mortality, especially in low- and middle-income countries. Early recognition of sepsis is crucial and sepsis should be treated by resuscitation with fluids and effective intravenous antibiotics should be given within one hour of the diagnosis [1]. The “golden hour of sepsis” stresses the relationship between timely initiation of antibiotic treatment and outcome: each hour delay in treatment reduces sepsis survival by 7.6% [2]. Pregnancy and delivery predispose women to infectious complications due to immunological and physiological alterations or from tissue damage during delivery. Recognition of sepsis during pregnancy, delivery and postpartum is difficult because of physiological adaptations to pregnancy, blood loss and increased maternal activity during labour [3]. WHO recently launched a new consensus defining maternal sepsis: a life-threatening condition defined as organ dysfunction resulting from infection during pregnancy, childbirth, post-abortion, or postpartum period [4].

Although the ‘golden hour of sepsis’ principle is not validated for women with pregnancy or in the puerperium due to a lack of studies, the principle is assumably even more important in pregnant, predisposed women where recognition is more difficult.

Globally, maternal sepsis (10%) is the third most frequent cause of direct maternal deaths, preceded by hemorrhage (27%) and hypertension (14%) [5–7]. In low- and middle-income countries (LMIC) maternal sepsis is a larger contributor to maternal mortality than in high-income countries (10.7% vs. 4.7% respectively) [5,8]. In high-income countries, however, maternal morbidity and mortality due to sepsis is increasing [9,10].

Suriname is an upper middle-income country in South America with a maternal mortality ratio of approximately 130 per 100.000 live births between 2010 and 2014 [11]. A confidential enquiry in Suriname in 1991 reported sepsis to be the third most frequent underlying cause of maternal death (n = 10/64, 16%) [12]. We recently published an increase in maternal deaths from sepsis, with sepsis as the most frequent cause (n = 17/65, 27%) [11]. This is poorly understood; therefore an in-depth case analysis was considered necessary.

Classification of maternal deaths into direct (obstetric) and indirect (non-obstetric) causes has given the impression that direct maternal deaths should receive greater attention than indirect deaths. However, since the focus is on the reduction of all preventable deaths, division between direct and indirect maternal deaths can be seen as arbitrary and counterproductive [13]. In this study, we therefore choose for a more theme-based approach by analysis of all sepsis-related deaths.

Primary objective of this study was to perform a qualitative analysis of the clinical aspects and management (based on the golden hour principle) of sepsis-related maternal deaths in Suriname. Secondary objectives were first to describe incidence and characteristics, second to analyze underlying causes, and third to evaluate quality of care and finally substandard care identification with audit to improve sepsis prevention, recognition and treatment strategies.

## Methods

Suriname is multi-ethnical with 541.638 inhabitants and one of the smallest populated countries in South America, with a density of 3,3 inhabitants per square kilometer. There are approximately 10.000 deliveries annually of which most in hospitals led by midwives and obstetricians (82%) [11]. Women with high-risk pregnancies are referred by the primary health services, which can take more than two hours, as some rural areas are only accessible by boat or airplane. Postpartum, women are usually discharged from hospital six hours after an uncomplicated delivery. They are seen at outpatient clinics or hospitals once, seven days after discharge. Postnatal care home visits are not done.

In 2015 a reproductive age mortality survey (RAMoS) was performed to identify maternal deaths in Suriname between 2010 and 2014 [11]. Medical records were collected of pregnancy related deaths identified by vital registration, or by screening of medical archives of all hospitals and primary care facilities. An anonymous case summary was made conform the FIGO-LOGIC *MDR clinical summary form* tool [14]. A local expert committee consisting of obstetricians, midwives, internal medicine specialists or anaesthesists reviewed each case summary. The committee agreed on the underlying causes and classified the cases [11]. To analyse substandard care factors an adapted version of the FIGO-LOGIC MDR *Grid analysis of clinical case management* form was used [14]. For this study specifically, medical records of all maternal deaths related to sepsis, were scrutinized for signs of sepsis, clinical management, primary sources of infection and causative pathogens. Data were manually entered into IBM SPSS version 21.0 (Armonk, New York, USA) for analysis. Descriptive statistics and frequencies were used to describe patient demographics, clinical and pregnancy characteristics and substandard care factors. Graphs were manually made in IBM SPSS version 21.0 and Microsoft Excel 2016 to demonstrate qualitative information on sepsis diagnosis and management.

### Definitions

Sepsis-related maternal deaths included deaths with sepsis as the underlying cause, sepsis as the mode of death and sepsis as a contributing factor. The underlying death cause was defined as the disease or condition that initiated the chain of events leading to death [15]. The mode of death was the disease or condition ultimately leading to death [15]. A contributing factor was defined as a condition existing before or developed during the chain of events leading to death, that predisposed the woman to death but was not causing death [15].

Clinical diagnosis of severe maternal sepsis was made by using the UK Obstetric Surveillance System definition, which is an adapted version of the systemic inflammatory response syndrome (SIRS) criteria: an assumed or proved infection with at least two of the four criteria (temperature of > 38°C or < 36°C, heart rate of > 100 beats per minute, respiratory rate of > 20 per minute, white blood cell count of > 17 x 10^9^ cells/L or < 4 x 10^9^ cells/L) measured on two occasions at least four hours apart [9,10]. Severe sepsis was associated with organ dysfunction (i.e. cardiovascular, respiratory, renal, coagulation, hepatic, neurological and uterine), hypoperfusion or hypotension [16,17]. Organ dysfunction was determined with the WHO near-miss tool [18]. In depth analysis of the maternal deaths with sepsis as underlying cause was performed in this study by determining when the first clinical signs of sepsis were manifested. The ‘golden hour’ principle (intravenous antibiotics given within an hour of severe sepsis diagnosis) was then evaluated [1].

Substandard care was defined as care below expected standards in the specific setting the woman was treated. The local expert committee evaluated substandard care in the absence of guidelines on sepsis in Suriname. Assessment of delay in receiving care was made by evaluating vital signs monitoring, diagnosing sepsis and initiation of antibiotic treatment. Other substandard care factors such as miscommunication, availability and patient-associated factors were also evaluated.

### Ethical considerations

The medical ethical review board of the Surinamese Central Committee on Research Involving Human Subjects and the Ministry of Health of Suriname approved the study [VG 006–15]. Patient’s names, hospitals and health care workers information remained confidential. No informed consent was required as only retrospective anonymized information from medical records of deceased women was used.

## Results

**Fig 1:**
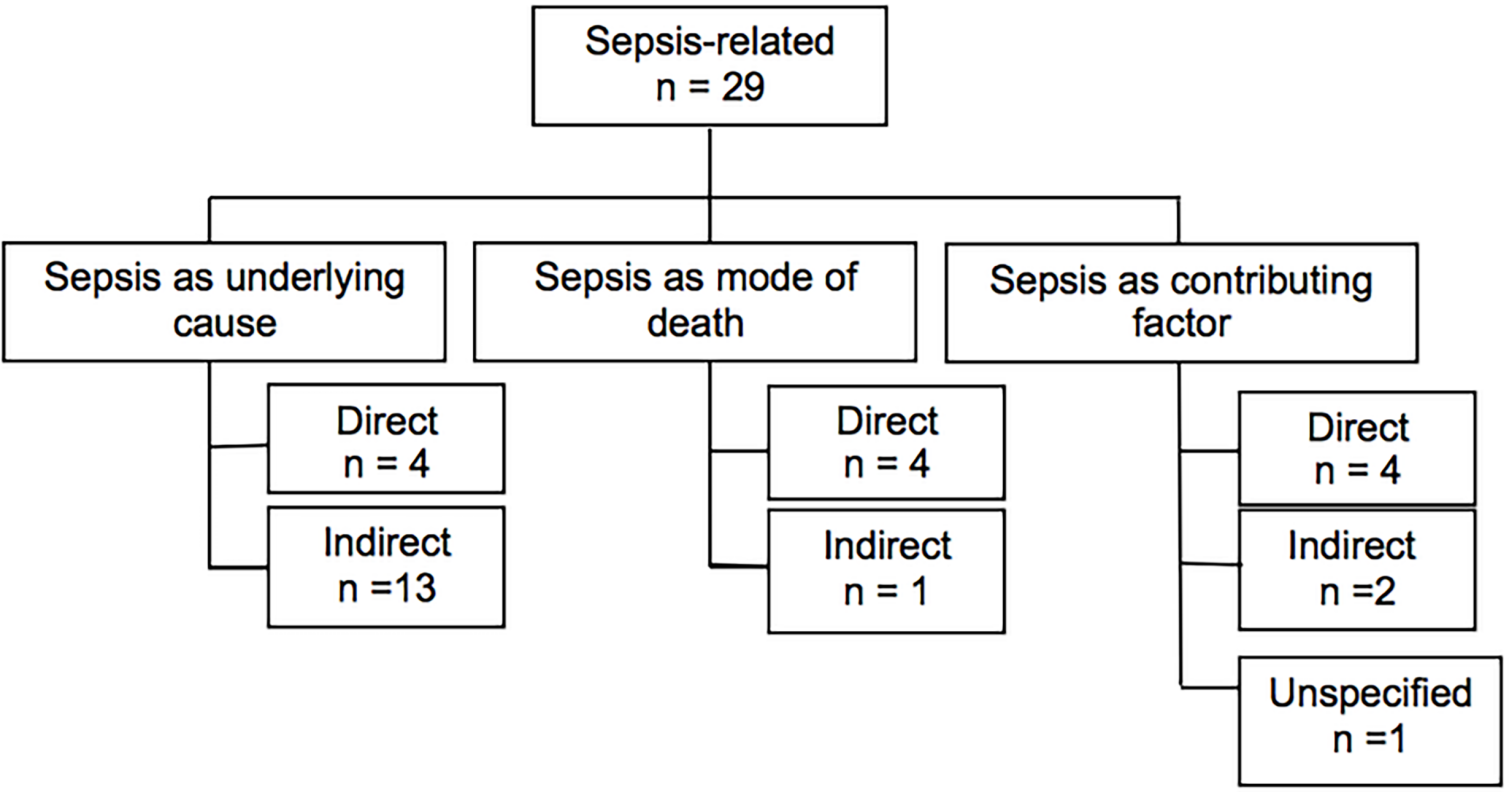
Overview of the sepsis-related maternal deaths between 2010 and 2014 in Suriname.

In the previously reported study on maternal mortality in Suriname between 2010 and 2014 sepsis was the most frequent underlying cause of death occurring in 17 of the 65 maternal deaths [11]. Of the women who died of other underlying causes, in five sepsis was the mode of death and in seven women sepsis was contributing to the death. Hence, in total 29 (45%) of the 65 maternal deaths were sepsis-related. (Fig 1) Medical records of two sepsis-related deaths (classified as indirect deaths with sepsis as underlying cause) were missing, therefore in-depth analysis of clinical aspects and substandard care was performed in 27 (93%) of sepsis-related maternal deaths. All the sepsis-related cases defined by the expert committee were also diagnosed by the clinicians who were in charge of the patients.

### Characteristics

In fourteen (48%) of the twenty-nine sepsis-related maternal deaths women were from maroon ethnicity, of which 13 (93%) had social insurance (insurance paid by the government for people of low socio-economic status) (Table 1). Death occurred postpartum in 21 women (72%), mostly within one week (n = 13, 62%). Two of the HIV-positive deceased women also had sickle cell (type SS) disease. Eighteen women (62%) died in the intensive care or coronary care unit, while nine (31%) died on the ward where critically ill women could not be monitored adequately. One woman died in the emergency department and one at home. Caesarean section was performed in eight (38%) of the 21 postpartum, sepsis-related deaths. All were elective cesarean sections; in two of these eight women the death was classified as a direct maternal death (Table 2). In four women a caesarean section was performed because of pre-eclampsia, in one case because of fetal distress, one woman had a sickle cell crisis, and two women were in a critical condition due to heart failure and Shigella sepsis.

**Table 1:** Characteristics of the sepsis-related maternal deaths in Suriname 2010–2014.

	**Sepsis deaths n = 29 (%)**
**Age**	
< 20	7 (24)
20–35	18 (62)
> 35	4 (14)
**Ethnicity**	
Hindu	5 (17)
Creole	6 (21)
Maroon	14 (48)
Javanese	3 (10)
Indigenous	0 (0)
Mixed	1 (4)
**Insurance** (missing 1)	
Social insurance	23 (82)
State health	4 (14)
Private	1 (4)
**Parity** (missing 2)	
< 3	18 (67)
≥ 3	9 (33)
Antenatal care^*^ (missing 6)	
None	4 (19)
< 4	6 (29)
≥ 4	11(52)
**Sickle cell disease** (missing 10)	
AS SS Negative	1 (5) 4 (21) 14 (74)
**HIV** (missing 6)	
Positive	6 (26)
Negative	17 (74)
**Anemia** in mmol/L (missing 6)	
No anemia	8 (35)
Severe anemia (Hb <4.3)	2 (9)
Moderate anemia (Hb 4,3–6,1)	10 (43)
Mild anemia (Hb 6.2–6.8)	3 (13)
**Mode of delivery (n = 21)**	
Spontaneous	13 (62)
Caesarean section	8 (38)
**Twins** (missing 6)	
Yes	3 (13)
No	20 (87)
**Perinatal death**^**#**^ (missing 2)	
Yes	13 (48)
No	14 (52)
**ICU-admission**	
Yes	18 (62
No	11 (38)
**Gestation at death**	
Early pregnancy	2 (7)
Antepartum (n = 6)	
< 28 weeks	2 (7)
28–33^+6^ weeks	2 (7)
≥ 34 weeks	1 (3)
GA unknown	1(3)
Postpartum (n = 21)	
≤ 48 hours	4 (14)
2–7 days	9 (31)
1–6 weeks	8 (28)

*Antenatal care starting from gestation of 16 weeks. (n = 27)

^#^ Perinatal death is death of fetus with a gestation of more than 22 weeks or 500 grams

**Table 2:** Case description of direct maternal deaths between 2010 and 2014 in Suriname with sepsis as underlying cause.

Case	Diagnosis	Culture	Time Death	Time to death*	ICU	Antibiotics
16 years Para 1 GA 37	Endometritis after manual placenta removal	Not performed	Postpartum 3 days	3 days	No	Amoxicillin orally
41 years Para 3 GA 36	Caesarean due to pre-eclampsia, complicated by endometritis and pneumonia	Blood culture: no growth	Postpartum 6 days	7 days	Yes	Initially amoxicillin orally. After 2 days intravenous amoxicillin, gentamycin and metronidazole
22 years Para 2 GA 39 HIV +	Vacuum extraction and episiotomy complicated by endometritis and a pneumonia	Blood culture: P. aeruginosa (no suscep-tibility done)	Postpartum 7 days	2 days	No	Initially ciprofloxacine orally profylactic. 5 days later intravenous cefotaxime, metro-nidazole, cotrimoxazole
28 years Para 4 GA 36 HIV + HbSS	Caesarean due to pre-eclampsia, complicated by a wound infection	Blood culture: no growth	Postpartum 7 days	2 days	Yes	Initially amoxicillin orally. After 5 days amoxicillin intravenously + metro-nidazole, gentamycin and ciprofloxacine

* Time between recognition of sepsis and death

### Classification and causes

#### a. Sepsis as the underlying cause

Four women with sepsis as underlying cause were classified as direct maternal deaths (Table 2). Underlying causes were endometritis in three women and wound infection in one woman. All women had term or near-term pregnancies and died within the first week postpartum. They had ruptured membranes less than 12 hours before delivery and none of the neonates died or showed signs of infection. The remaining 13 deaths were classified as indirect maternal deaths: pneumonia (n = 7, 54%), meningitis (n = 2), gastro-enteritis (n = 2), urosepsis (n = 1) and HIV therapy-induced hepatitis (n = 1).

#### b. Sepsis as the mode of death

These five cases included death from 1) a bowel perforation following a mechanically induced abortion; 2) a central venous line sepsis in a woman in the ICU with bleeding from coagulation disorders following fetal death syndrome; 3) a craniotomy wound infection in a hypertensive woman with intracranial bleeding and eclampsia; 4) severe sepsis following multi-organ failure after iatrogenic hypotension due to overdose of antihypertensive medication in severe pre-eclampsia and 5) endocarditis in a woman with aortic valve prosthesis.

#### c. Sepsis as a contributing factor

In seven cases sepsis was a contributing factor; underlying causes were severe pre-eclampsia / eclampsia (n = 4), diabetic kidney failure with an infected diabetic foot and osteomyelitis (n = 1), heart failure in a woman with mitral valve prostheses and endocarditis (n = 1) and one case where sepsis contributed to the death but with the cause remaining unclear.

The main cause of infection was pneumonia, which affected 14 women (48%), followed by wound infections (n = 3, 10%) and endometritis (n = 3, 10%). Blood, urine and/or sputum cultures or vaginal swabs were obtained in 23 cases (85%). No culture was done in four cases because of temperature below 38 degrees (n = 2), very rapid deterioration of the condition of the patient (n = 1) and loss of blood sample before reaching the laboratory (n = 1). Results of the cultures were available in 20 cases and not traceable in the remaining three cases. Either one of the cultures were positive in 15 cases (75%). Blood culture showed growth of pathogens in 10 cases (50%) (Table 3).

**Table 3:** Micro-organisms isolated from the cultures performed in the sepsis-related maternal deaths in Suriname.

Blood cultures positive n = 10/20 (50%)	Urine cultures positive n = 3/13 (23%)	Sputum cultures positive n = 4/6 (66%)
**Pseudomonas aeruginosa[n = 2]**	Actinobacter	Pseudomonas aeruginosa [n = 2]
**Enterobacter [n = 2]**	Klebsiella pneumoniae	Klebsiella pneumoniae [n = 2]
**Gram negative rods [n = 2]**	Escherichia Coli	
**Shigella flexneri**		
**β-hemolytic streptococcus group A**		
**Enterococcus faecalis**		

### Clinical aspects

**Fig 2:**
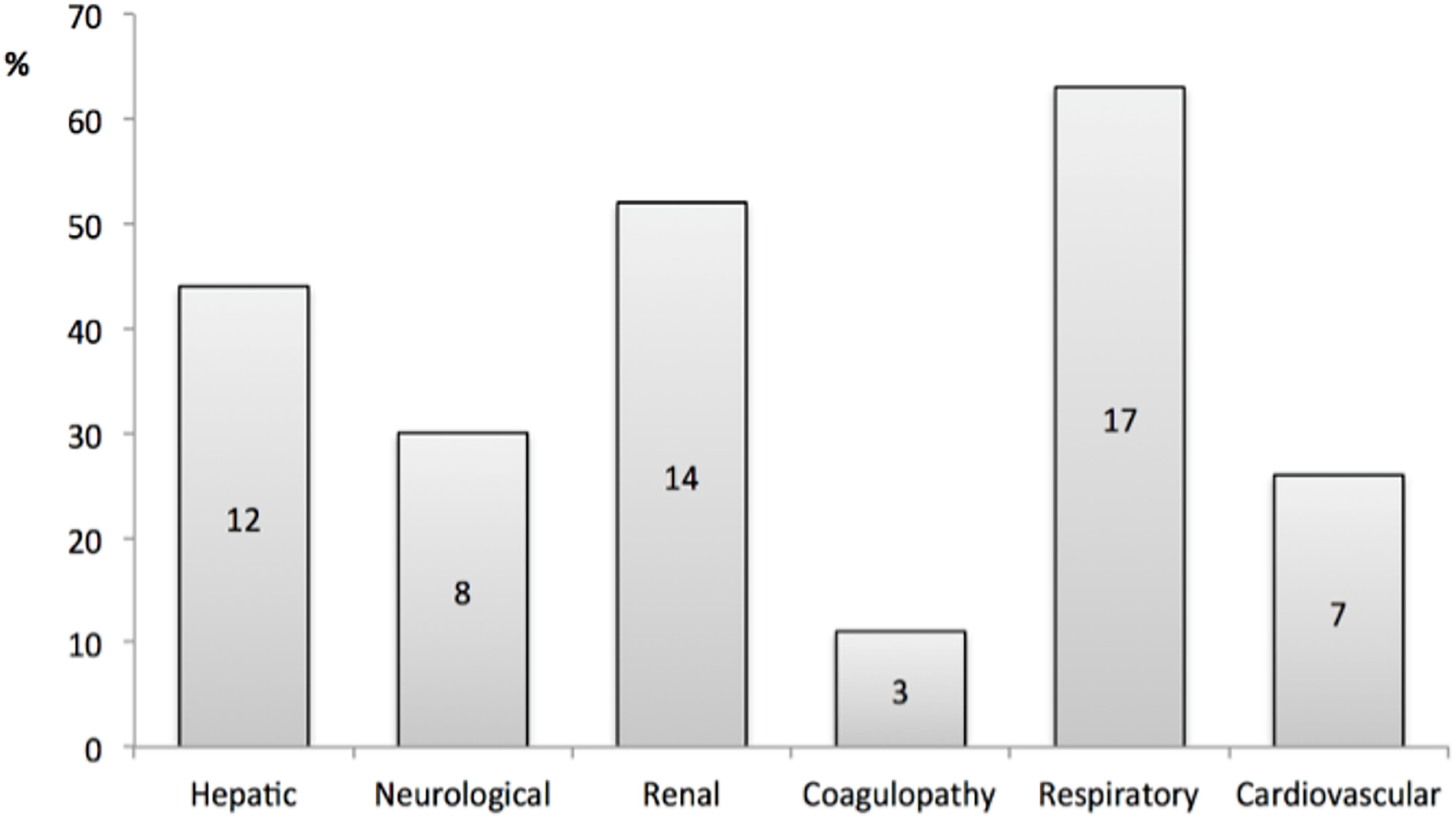
Number of sepsis-related maternal deaths showing different organ system dysfunctions (n = 27).

Fig 2 demonstrates the number of cases per dysfunctioning organ system. At least two organ dysfunctions were present in 20 (74%) cases. The respiratory system was the most frequently documented organ dysfunction in 17 cases (63%), followed by the renal (n = 14, 52%) and hepatic system (n = 12, 44%).

### Substandard care

Substandard care factors which contributed to death were identified in 25 women (93%). Delay in reaching care occurred in four women (15%), while delay in receiving care in the hospital occurred in 24 women (89%) (Table 4).

**Table 4:** Substandard care analysis of sepsis-related maternal deaths in Suriname between 2010 and 2014.

	Sepsis-related deaths n = 27 *(100%)
**Delay in reaching care**	4 (15)
**Delay in receiving care**	24 (89)
**Health care provider factors**	
Insufficient quality of care	21 (77)
Inadequate monitoring	16 (59)
Poor Communication	10 (37)
Delay in diagnosis	17 (63)
**Unavailability**	
Staff	2 (7)
Medication	4 (15)
**Patient factor**	
Poor adherence to medication	7 (26)
**Substandard factors leading directly to maternal death**	
Definitely / most probably	10 (37)
Possibly	13 (48)
Unable to determine	2 (7)

* 27 sepsis-related deaths could be analysed in-depth due to 2 missing files

#### Delay in monitoring & diagnosis

**Fig 3:**
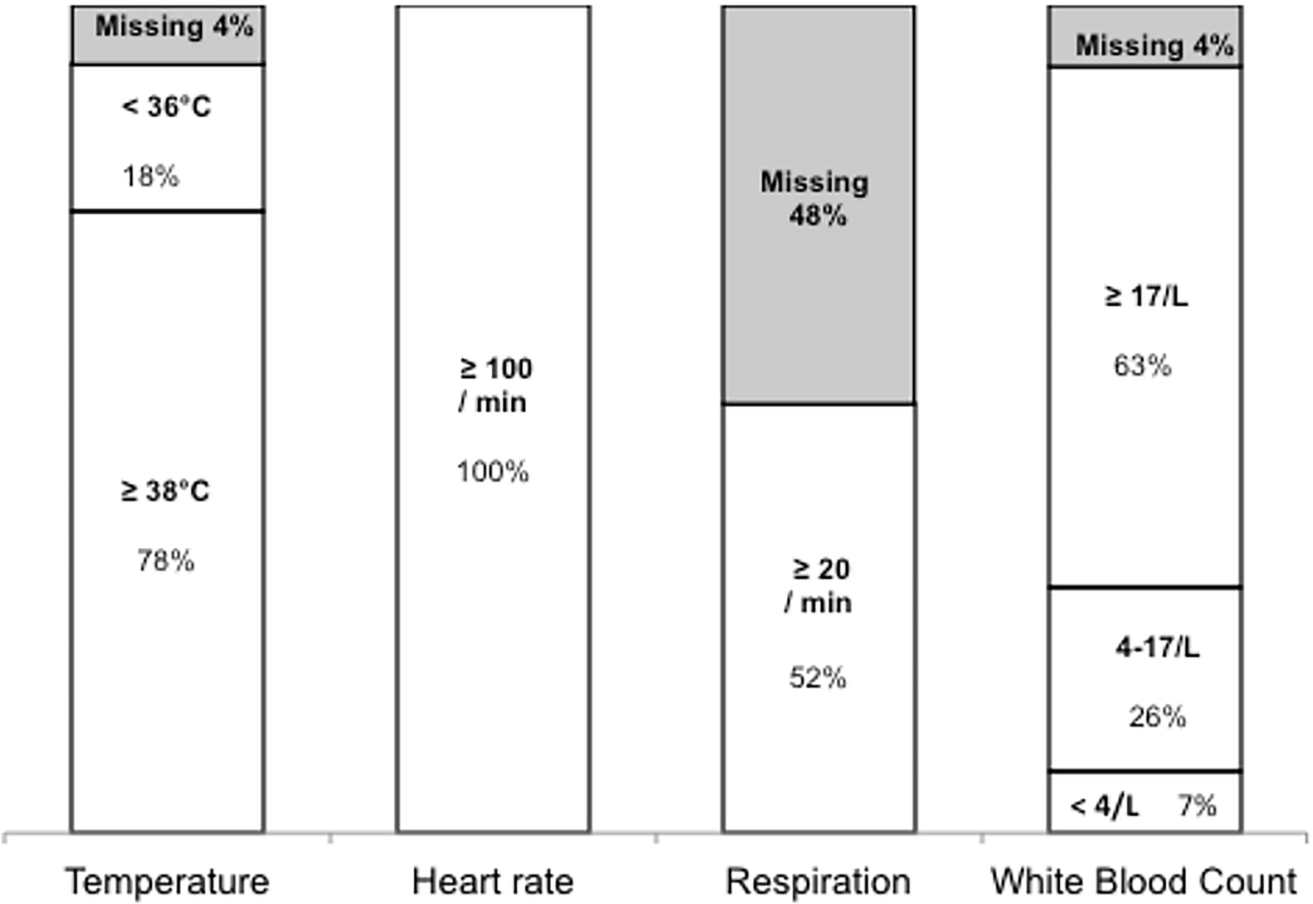
Clinical signs when sepsis was diagnosed in the sepsis-related maternal deaths in Suriname between 2010 and 2014 (n = 27).

The expert committee identified delay in the diagnosis of sepsis in 17 women (63%). Inadequate monitoring occurred in 16 women (59%). In Fig 3 the adapted SIRS-criteria that were used and reported by clinicians in the 27 cases are shown. Respiratory rate was the most poorly reported vital sign, reported in only 13 women (52%). Temperature was below 36 degrees in five women (18%) and white blood cell count was normal in seven women (26%). Information on mental state was missing in 19 women (70%).

**Fig 4:**
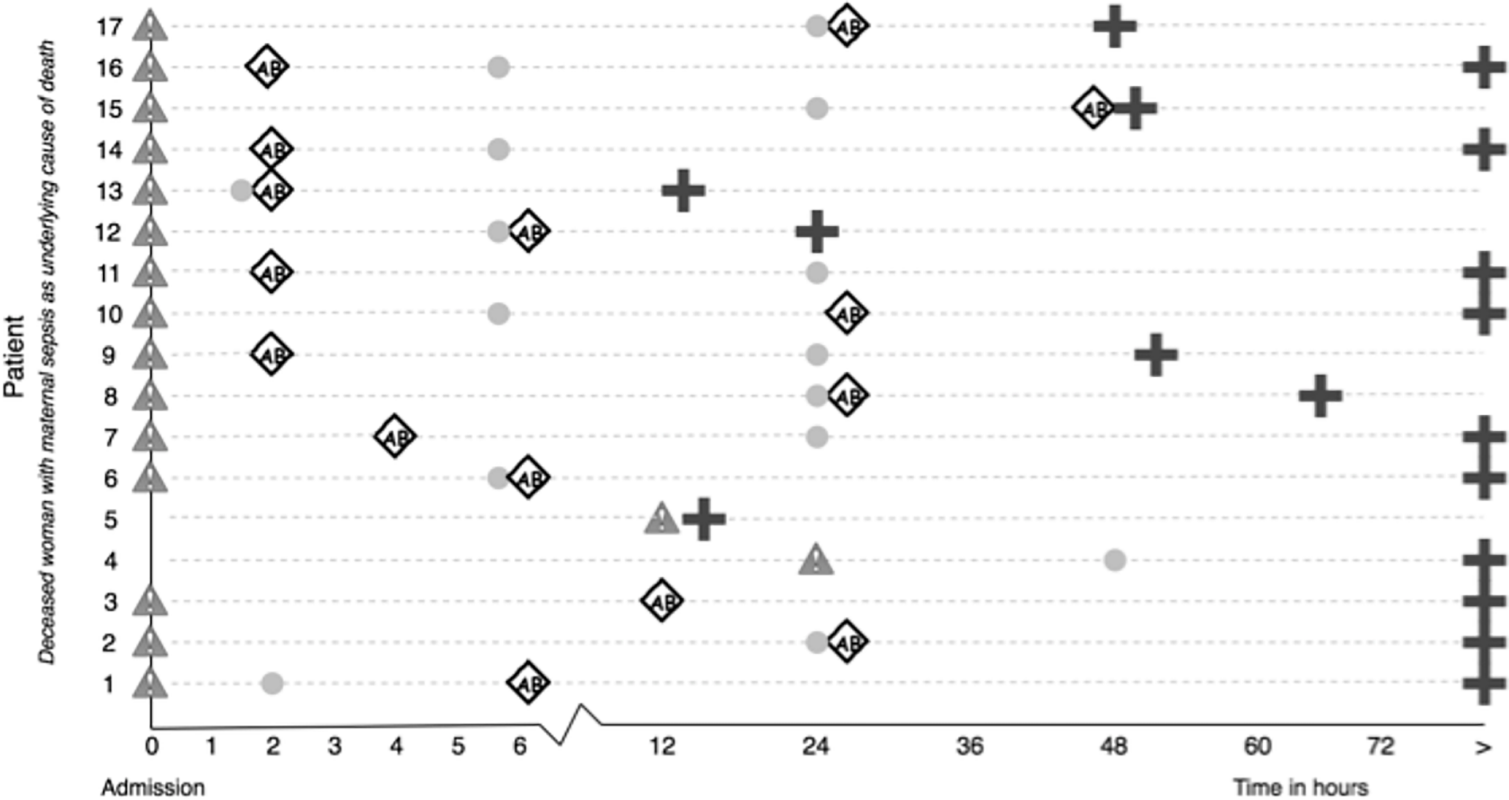
Overview of the time between admission, first signs of sepsis, first vital signs after admission, the initiation of antibiotic treatment and death per patient with sepsis as the underlying cause of death (n = 17). Legend: initial signs of sepsis designated with a gray triangle, use of antibiotics designated with grey rhombus, death designated with a black cross, vital signs recorded after initial signs of sepsis designated with a grey circle.

In-depth analysis of the maternal deaths with sepsis as the underlying cause (n = 17) is provided in Fig 4 and the supplementary files S1 Table and S1 Fig. Vital signs were taken upon admission in all cases, though any of the vital signs were rechecked within 24 hours in only seven septic women. According to documentation temperature, pulse and blood pressure were rechecked within 24 hours in respectively four (24%), five (30%), and six (35%) women. Organ dysfunction was already present when initial signs of sepsis were manifest in 15 of the 17 women. In two women no information was available because no laboratory tests were done at the time sepsis was diagnosed.

#### Delay in treatment and the golden hour principle

The committee agreed that there was delay in treatment in 22 women (81%). Intravenous antibiotic treatment was given in 25 of the 27 women (93%). In 12 women (44%) empiric antibiotic treatment appeared to be right according to the culture sensitivity profiles. In eight women (30%) frequent switch in antibiotics, with more than three different regimes, was given within three days, without sensitivity profiles known. In-depth analysis of the maternal deaths with sepsis as the underlying cause (n = 17) illustrated that 15 women (88%) had already signs of severe sepsis when admitted in the hospital. In none of those women antibiotics were administered within the first hour of diagnosis of sepsis (Fig 4). Mean (SD) time between the first sign of sepsis and initiation of intravenous antibiotic treatment was 12.5 hours (SD 5, range 2–48 hours). In five (29%) women intravenous antibiotics were administered more than 24 hours after the onset of sepsis. No intravenous antibiotics were administered in two women: in one case the woman died within two hours after the diagnosis and in the other woman antibiotics were given orally.

#### Other substandard care factors

Delay due to miscommunication between health care professionals occurred in ten cases (37%) (Table 4). An example where miscommunication occurred is the case of direct maternal death from sepsis after manual placenta removal: one gynaecologist prescribed primperan (a gastrointestinal stimulant) for vomiting in this women with a bumped and shiny belly and another stopped it the next day considering primperan to be contra-indicated when an intestinal obstruction is suspected. In three cases (11%) an Intensive Care bed was requested but not available. In one case (4%) blood was not available for transfusion. The expert committee agreed that substandard care factors definitely or most probably led to death in 10 of the 27 women (37%).

## Discussion

This is the first detailed clinical study of pregnant and postpartum women dying of sepsis in Suriname. Of all 65 maternal deaths from 2010–2014 in Suriname 29 (45%) were sepsis related and in 17 of these women (27%) sepsis was the leading underlying cause of death. The attribution of sepsis to maternal deaths in Suriname was much higher than the 8.3% reported in Latin America and Caribbean or the 10.7% worldwide [5]. In Brazil, however, infection was responsible for nearly half (46%) of all facility-based maternal deaths, much higher than previously thought [19]. While various high-income countries performed extensive qualitative studies on sepsis-related maternal mortality and morbidity, there is scarce data from middle- or low-income countries [9,10,19–22].

Three major findings of our study were identified: first, most sepsis-related maternal deaths occurred in women with low economic status and postpartum, within one week after delivery; second, the most common identified source of sepsis causing maternal deaths in Suriname was pneumonia; and finally, there was a major delay in monitoring, diagnosis and prompt treatment with regards to the golden hour principle.

### Classification and causes of sepsis-related maternal deaths

Classifying the cause of maternal death is a complex matter with great classification differences between countries [23]. WHO guideline for ICD-MM classification states that the underlying cause of maternal death is where the chain of events leading to death starts. The ICD-MM classification system, however, impedes for example “a death with an abortive outcome” to be classified as a “pregnancy-related infection”. Also, a woman can only have one underlying cause of death for classification purposes. One of the cases in our study, a woman who died due to complications (sepsis) of a mechanically-induced abortion, was classified as “a death with an abortive outcome”, would not be included in this study if only underlying causes were studied. Similarly, another case of a woman with cerebral bleeding due to hypertensive disorder who died due to a sepsis caused by the craniotomy wound, would not have been included (sepsis as mode of death).

While malaria in pregnancy caused maternal mortality in the nineties in Suriname (4,7%, n = 3/64), no maternal deaths due to malaria have been diagnosed between 2010 and 2014.[11,12] This is in line with the numbers in the general population: deaths from malaria have declined with 92% since 1990 [24].

Pneumonia was the most common source of sepsis in Suriname. Accordingly, as in the UK, not only genital tract sepsis but more importantly non-obstetric causes as especially pneumonia, but also urosepsis were reasons for maternal mortality [22]. The attribution of indirect causes has been increasing globally [5]. An improved enquiry and registration of deceased women on non-obstetric wards, as done in this study, can also be the result of the relative increase of indirect maternal deaths in middle-income countries. Because a RAMoS was done nationwide, also indirect deaths at other wards than the maternity ward were included, leading to more non-obstetric cases as pneumonia [11].

### Substandard care

Delays in reaching health care facilities were not a major problem (n = 4/27, 15%). Each year only 5% (n = 500/10.000) of deliveries take place in the rural interior and these are mainly low risk pregnancies. However, delays in receiving quality care in health facilities occurred more frequently (n = 24/27, 89%): there was delay in monitoring, diagnosis and treatment of sepsis-related deaths [25]. Suriname, with a MMR of 130, could be classified as stage III in the WHO model of “obstetric transition”, which describes the shift of countries from high MMR to low ratios [25]. In this stage of transition indirect causes such as non-obstetric sepsis, are becoming important contributors to maternal deaths, whereas direct maternal deaths still remain significant. In this model essential recommendations to reduce maternal mortality for stage III include improvement of quality of intra-hospital care (third delay), with skilled birth attendance and appropriate management of complications [25,26]. Therefore, we focus on these delays in health facilities in greater detail.

#### Monitoring

Adequate monitoring of pregnant women for clinical signs of infection in early stages is crucial [27,28]. To identify critically ill pregnant women a modified early obstetric warning score (MEOWS) could be used [29]. To perform MEOWS systolic blood pressure, diastolic blood pressure, respiration rate, heart rate, oxygen saturation, temperature and conscience level should be assessed repeatedly. Recognition of predetermined abnormal values of these vital signs should lead to an adequate medical response [29]. In this study substandard care by poor monitoring occurred in 16 of 27 women (59%) and there had been inadequate recognition of early warning signs. No structural scoring of vital parameters as the MEOWS was used. In all sepsis-related cases initially there was a tachypnea of more than 20 respirations per minute (documented in 48% of the records) and / or a tachycardia of more than 100 per minute indicating first signs of severe sepsis [20]. However, these early signs of sepsis were not recognized in nine women (32%) as they died on the ward without receiving adequate monitoring and treatment.

Clinical characterization of sepsis may be achieved by performing a SOFA (sepsis-related or sequential organ failure assessment) score, which determines the extent of organ dysfunction [30,31]. Though SOFA is not validated in pregnant women, a simplified form of SOFA, the quick SOFA or qSOFA (respiration rate ≥22 / minute, altered mentation and systolic blood pressure < 90 mm/Hg) can be used as a simple bedside test to identify women with suspected infection associated with poor outcome. Respiratory rate also seems to correlate with severity of sepsis [27]. In this study we did not use qSOFA as diagnostic or prognostic criterium as it is not validated in pregnant women. More importantly information on respiration rate (n = 13, (48%)) and mentation (n = 19, 70%) were often missing in our population and it was therefore not possible to assess qSOFA scores.

#### Diagnosis and treatment

This study illustrated that delay in monitoring led to delay in diagnosis and treatment of sepsis. Even when sepsis was recognized, in none of the cases antibiotic treatment was started within one hour. According to the Surviving Sepsis Campaign guidelines any sign of infection should promptly be recognized and treated [1]. Aggressive fluid resuscitation and early and appropriate antibiotic treatment is the best way to manage sepsis [1,3]. Antibiotic treatment should be started within one hour (golden hour principle) [1].

### Recommendations to prevent maternal deaths from sepsis in Suriname

From this maternal death from sepsis analysis we could distillate three major recommendations for maternal care in Suriname: 1) improve postpartum care, 2) introduce a maternal sepsis bundle for diagnosis and 3) early treatment of pregnant and postpartum women in close collaboration with other medical disciplines.

Since most deaths occurred after delivery, it is vital to provide women with sufficient information of danger signs when discharged after delivery. Furthermore, the initiation of a structured postpartum care system in Suriname is crucial.

The introduction of a structured recording of vital signs (as MEOWS) is strongly recommended in order to identify critically ill septic patients[29]. Sepsis performance improvement programs which includes guidelines on monitoring, prevention and early treatment of sepsis are necessary [1]. Introduction, implementation and adherence to Surviving Sepsis Campaign (SSC) bundles (a set of recommendations for sepsis screening and treatment) could enhance the care for septic pregnant and postpartum women in hospitals. Selection of an optimal intravenous empiric antimicrobial regimen is the cornerstone of the treatment of sepsis [1]. A nationwide guideline should be developed and implemented in Suriname.

As non-obstetric causes of sepsis are becoming more important, a multidisciplinary approach in treatment of sepsis is essential [1,22]. Collaboration of obstetricians with other physicians as internal medicine specialists, microbiologists, nurses, and pharmacists is mandatory.

### Strengths and limitations

Regarding the difficulties collecting clinical data from medical records in a middle-income country, this extensive dataset is unique and valuable. There are, however, some limitations. Cases were analysed and classified by the expert committee based on information of medical records, in which documentation was not always sufficient and sometimes information was missing. However, the local team was accustomed to these records and scrutinized all medical information for signs of recognized medical comorbidities predisposing pregnant and postpartum women to infection including obesity, diabetes mellitus, HIV / AIDS, hepatitis, sickle cell disease, malaria, malnutrition, multiple gestations and severe anemia [1,21,32]. Unfortunately, in this study information on weight and nutrition of the women was not available. At the moment we are prospectively collecting morbidity data for all pregnant women in Suriname.

Finally, while WHO launched the new definition of maternal sepsis, it remains difficult to compare data between countries because various criteria and definitions are used [4]. The WHO GLOSS, the Global Maternal Sepsis Study, in more than 500 healthcare facilities in 53 countries will address these issues [4].

## Conclusions

Sepsis was the leading cause of maternal death in Suriname, with most deaths occurring after delivery. Non-obstetric causes (as pneumonia) were the most important primary contributors to sepsis. Monitoring of critically ill septic patients was inadequate and antibiotics were not started within the “golden hour”. A uniform international definition of sepsis in pregnancy / postpartum with clear criteria is mandatory for early recognition of sepsis. Close monitoring and prompt treatment of patients with sepsis is essential. Introduction of early sepsis warning signs, guidelines on postpartum care and introduction and implementation of SSC bundles for pregnant and postpartum women could prevent maternal deaths from sepsis.

## Supporting information

S1 Table Vital parameters in the 17 cases with sepsis as underlying cause. (XLS)

S1 Fig Figures of the vital parameters in the 17 cases with sepsis as underlying cause. (XLSX)
